# Hydrolyzable Tannins in the Management of Th1, Th2 and Th17 Inflammatory-Related Diseases

**DOI:** 10.3390/molecules27217593

**Published:** 2022-11-05

**Authors:** Stefano Piazza, Marco Fumagalli, Giulia Martinelli, Carola Pozzoli, Nicole Maranta, Marco Angarano, Enrico Sangiovanni, Mario Dell’Agli

**Affiliations:** Department of Pharmacological and Biomolecular Sciences, University of Milan, 20133 Milan, Italy

**Keywords:** hydrolyzable tannins, ellagitannins, gallotannins, inflammation, Th1, Th2, Th17, rheumatoid arthritis, inflammatory bowel diseases, atopic dermatitis

## Abstract

Plants rich in hydrolyzable tannins were traditionally used all over the world for a variety of chronic inflammatory disorders, including arthritis, colitis, and dermatitis. However, the knowledge of their immunological targets is still limited though fundamental for their rational use in phytotherapy. The recent advances regarding the pathogenesis of inflammatory-based diseases represent an opportunity to elucidate the pharmacological mechanism of plant-derived metabolites with immunomodulatory activity. This review collects recent articles regarding the role of hydrolyzable tannins and their gut metabolites in Th1, Th2, and Th17 inflammatory responses. In line with the traditional use, rheumatoid arthritis (RA), inflammatory bowel diseases (IBDs), psoriasis, atopic dermatitis (AD), and asthma were the most investigated diseases. A substantial body of in vivo studies suggests that, beside innate response, hydrolyzable tannins may reduce the levels of Th-derived cytokines, including IFN-γ, IL-17, and IL-4, following oral administration. The mode of action is multitarget and may involve the impairment of inflammatory transcription factors (NF-κB, NFAT, STAT), enzymes (MAPKs, COX-2, iNOS), and ion channels. However, their potential impact on pathways with renewed interest for inflammation, such as JAK/STAT, or the modulation of the gut microbiota demands dedicate studies.

## 1. Introduction

**Figure 1:**
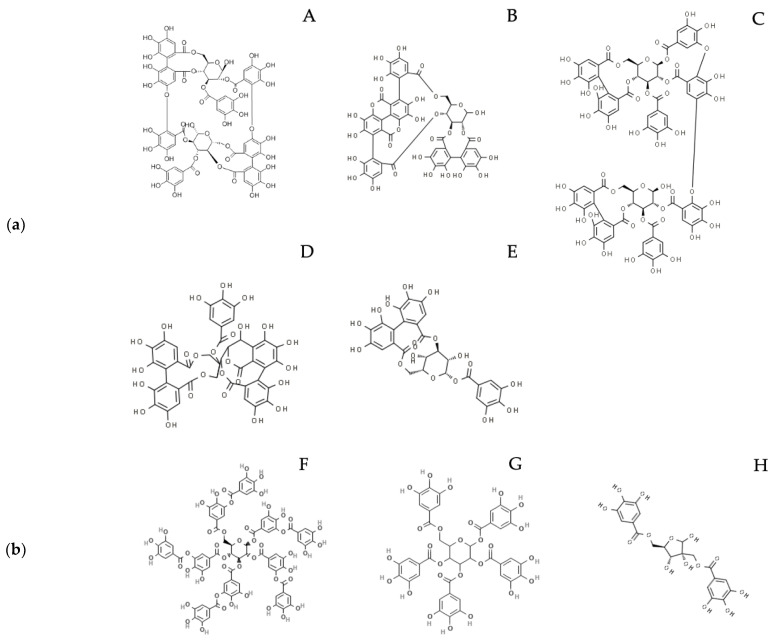
(**a**) Examples of ellagitannins cited in the main text: A. Oenothein B; B. Punicalagin; C. Tamarixinin A; D. Casuarinin; E. Corilagin. (**b**) Examples of gallotannins cited in the main text: F. Tannic acid; G. 1,2,3,4,6 Pentagalloyl glucose; H. Hamamelitannin.

**Figure 2:**
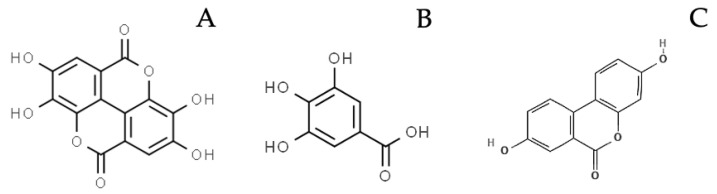
Examples of compounds deriving from HTs hydrolysis and gut metabolism, cited in the main text: A. Ellagic acid; B. Gallic acid; C. Urolithin A.

Hydrolyzable tannins (HTs) consist of gallotannins (GTs) and ellagitannins (ETs): the first are composed by a variable number of galloyl-moieties linked to a glycosylic unit, while the latter also contain hexahydroxydiphenic acid (HHDP) moieties (Figure 1a,b). After chemical or enzymatic hydrolysis, gallotannins release gallic acid (GA), while ellagitannins release also HHDP, spontaneously converted to ellagic acid (EA). GA and EA are available for absorption in their form or after gut microbiota metabolism, which transforms GA to pyrogallol derivatives and EA to urolithins (Figure 2) [1,2].

Plants containing HTs were reported by traditional medicine belonging to different geographical areas on the basis of their common astringent properties [3,4,5]. The ethnopharmacology from the Mediterranean area includes rich sources of GTs and ETs, such as pomegranate (*Punica granatum* L.) and other berries (*Fragaria* spp., *Rubus* spp.), walnut (*Juglans regia* L.), sumac (*Rhus coriaria* L.), sweet chestnut (*Castanea sativa* Mill.), and oak (*Quercus* spp.). Despite growing knowledge on the metabolism and pharmacological properties of HTs, their application for the control of inflammatory-based diseases is still limited. In 2018, Kiss and Piwowarski summarized the pre-clinical evidence regarding the anti-inflammatory activity of HTs and derived metabolites from various sources, with a particular focus on cardiovascular and inflammatory bowel diseases (IBDs) [6]. Similarly, our research group sustained the evidence regarding the role of berries in skin and gastro-intestinal inflammation [7,8,9,10]. This body of pre-clinical studies demonstrated that HTs and their metabolites reduce the release of innate inflammatory mediators (TNF-α, IL-6, IL-1β, PGE2, MMPs), mainly through NF-κB impairment.

Of note, the research for novel therapeutical strategies, often inspired by natural compounds, has been renewed by recent discoveries regarding the pathogenesis of inflammatory-based diseases, such as rheumatic disorders, inflammatory bowel diseases, psoriasis, atopic dermatitis (AD), and asthma. The number of publications on PubMed regarding immune-mediated inflammatory diseases increased from 309 to 1059 per year during the last 10 years. Cytokines involved in Th polarization and response, such as IFN-γ, IL-23, IL-17, and IL-4, represent renewed targets for novel pharmacological therapies [11]. Accordingly, the inhibition of their molecular signaling, with a particular reference to the JAK/STAT pathway, is an emerging strategy to control several inflammatory diseases [12].

The potential role of HTs against inflammatory-based diseases might also involve their impact on gut microbiota: generally speaking, these molecules have poor bioavailability and undergo extensive metabolism at the gut level, thus leading to the formation of small metabolites with prebiotic effects [6,8,13]. From this point of view, HTs can be considered potential pro-drugs and prebiotics; from another point of view, their poor absorption is exploitable to conceive an in situ action on human epithelia, with minor side effects at the systemic level. To this end, it is relevant to consider that several inflammatory-based diseases are triggered by the interaction among the environment and the immune system at the level of physiological barriers, such as respiratory, intestinal, and cutaneous.

On this basis, our review aims to update the evidence on the inflammatory targets of plant extracts rich in HTs, with a focus on Th1, Th2, and Th17 responses. For this purpose, the most common inflammatory diseases in which Th-derived cytokines play a key role in pathogenesis have been considered. Of note, the role of HTs in rheumatoid arthritis (RA), psoriasis, AD, and asthma was not recently revised. The bibliographic research took into consideration the crucial role of gut metabolism and bioavailability after oral consumption.

## 2. Results

### 2.1. Th1 and Th17 Inflammation: Rheumatoid Arthritis, Psoriasis, and IBDs

**Figure 3:**
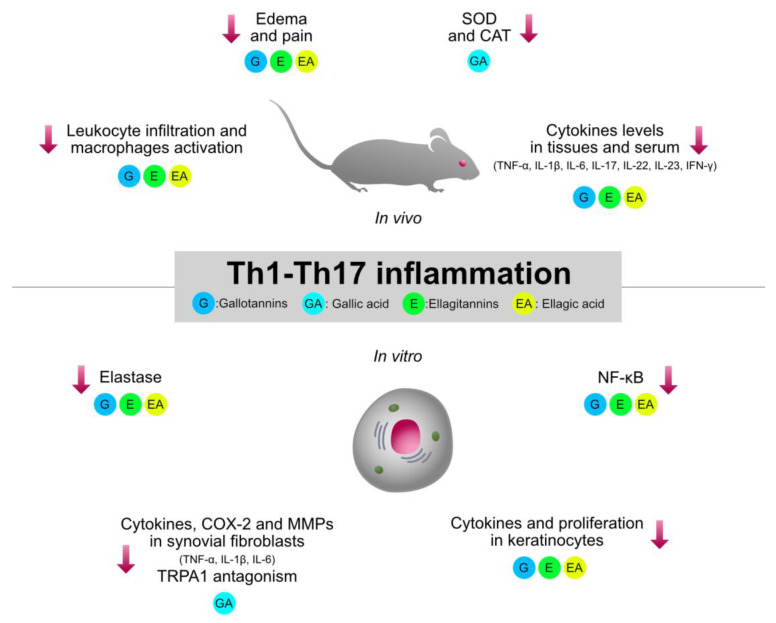
Graphical summary of the anti-inflammatory mechanisms of HTs and their hydrolysis derivatives (GA, EA), investigated in type 1 inflammation models (RA, IBDs, psoriasis). The compounds share immunosuppressive properties on innate cytokines (TNF-α, IL-1β, IL-6) and type 1 cytokines (IL-23, IL-17, IFN-γ), which are modulated by NF-κB and STAT pathways. G, gallotannins; E, ellagitannins; GA, gallic acid; EA, ellagic acid. Red arrows indicate inhibition.

Type 1 inflammation, orchestrated by Th1 cells, plays a crucial role in the pathogenesis of immune-mediated inflammatory diseases, such as rheumatoid arthritis (RA), IBDs, and psoriasis. Traditionally used HTs-containing plants and relative isolated compounds were investigated by several pre-clinical models involving type 1 inflammation. The collection of the experimental evidence allowed a better clarification of the structure–activity relationship regarding the immunomodulatory mechanisms shared by HTs, which are summarized in Figure 3.

#### 2.1.1. Rheumatoid Arthritis (RA)

The etiologies of RA, psoriasis, and Crohn’s diseases (CD) share several common pathogenetic pathways and are frequently associated as co-morbidities [14]. RA is a chronic autoimmune inflammatory disorder, influenced by genetic and environmental factors. Mediators from innate immunity (TNF-α, IL-6) and adaptive immunity, namely type 1 cytokines (IL-12, IL-17A, IFN-γ), are crucial for the pathogenesis of RA [15]. The main aim of pharmacological treatment is to relieve inflammation and to recover motor function to prevent long-term complications. Over the past decades, the knowledge regarding the pathophysiology of RA drove the discovery of several efficacious agents to cure patients unresponsive to DMARDs therapy, such as JAK/STAT inhibitors and novel biological drugs (i.e., anti-IL-17 and anti-IL-6 antibodies). However, the need for personalized medicine and the safety of chronic immunosuppressive treatments both remain a major issue [16].

Traditional medicine from different countries, including Mediterranean countries, is also based on plants containing HTs to attenuate articular pain and inflammation. Pomegranate (*Punica granatum* L.) is one of the most investigated dietary sources of ETs and free EA; this crop is widespread in the Mediterranean area, with an especially long tradition in Middle-Eastern countries. The pre-clinical evidence on the potential anti-inflammatory role of pomegranate for RA was recently reviewed by Mahdavi et al., in 2021 [17]; thus, only unreviewed articles or studies regarding the role of specific pomegranate tannins were included in the present review. The authors reported eight in vivo studies, mainly conducted on polar extracts from pomegranate peel (10–320 mg/kg) administered per os (p.o.) in a rodent model of Freund’s complete adjuvant (FCA)-induced arthritis. In three articles, homogeneous doses of extracts (10–50 mg/kg) were shown to decrease the arthritis score; the mechanisms that explain the biological effects include the inhibition of pro-inflammatory cytokines (IL-6, TNF-α) and the increase of antioxidant enzymes (MDA, GSH, SOD) [18,19,20]. Another in vivo study also demonstrated that the topical application of a standardized pomegranate peel extract can counteract the FCA-induced arthritis as much as 1% diclofenac. The inhibitory effect on edema and pain was observed at doses ranging from 1 to 5%, containing EA 0.13 to 0.65% w/w, respectively. The administration of EA alone was at least partially able to explain the bioactivity. Similarly, the histological analysis showed the reduction of leukocyte infiltration and cytokines levels (TNF-α, IL-1β) [21].

The bioactivity of pomegranate is often attributed to free EA and to its main ET, punicalagin. In vivo studies confirmed that the intra-peritoneal administration (i.p.) of punicalagin (10–50 mg/kg) inhibited joint edema in adjuvant-induced and collagen-induced models of arthritis [22,23,24]. In vitro experiments showed that punicalagin may act on M1 macrophages and fibroblast-like synoviocytes by inhibiting the NF-κB pathway and cytokine release (TNF-α, IL-1β, IL-6) [22,24]. From this investigation, punicalagin resulted in the most investigated pure ET for a potential anti-arthritis effect, although i.p. administration limited the speculation on the role of ET biotransformation at the gut level.

A similar limitation exists in the study from Zhuang et al., in which another ET, tamarixinin A (12.5 or 50 mg/kg/d), was administered via subcutaneous injection to collagen- or FCA-induced arthritis [25]. According to previous results, the compound showed the suppression of the progression and development of arthritis in both models. Again, tamarixinin A (5, 10, 20 μM) impaired the release of NO and cytokines (TNF-α, IL-6) in LPS-activated peritoneal macrophages; the mechanism of action was ascribed to NF-κB and p38 MAPK inhibition. Similarly, oenothein B (1–60 μg/mL, corresponding to 1.27–76.2 μM) inhibited cytokines (TNF-α, IL-1β, and IL-6) and iNOS expression in stimulated RAW 264.7 macrophages: the inflammatory model was TLR2- or TLR4-dependent, since selective agonists or LPS were used for the stimulation. Once again, the activity correlated with the inhibition of p65 and p50 (NF-κB) translocation. Oenothein B was unable to counteract IFN-γ-induced iNOS, thus excluding the interference with the IFN-γ pathway. Of note, the GA moiety was not responsible for the bioactivity, thus suggesting the importance of the whole chemical structure [26]. On the other hand, the plausibility of the interaction among unmetabolized ETs and circulating immune cells is poor, according to their low bioavailability. On the other hand, the potential anti-inflammatory role of low μM (<2.5 μM) oenothein B, isolated from *Myrtus communis* L., was also demonstrated in human gastric epithelial cells stimulated by TNF-α or IL-1β [27].

Sources of ETs other than pomegranate were less investigated. However, relevant information regarding the bioactivity of dietary products containing ETs comes from few studies performing oral administration. Javed et al. evaluated the effect of walnut (*Juglans regia* L.), in comparison to methotrexate (0.5 mg/kg), in a model of FCA-induced arthritis. The oral consumption of raw walnut (10% *w*/*w* of the total feed) or hydroalcoholic extract (900 mg/kg) increased the serum total antioxidant capacity (TAC) by elevating the antioxidant enzyme level (SOD, CAT). Moreover, RA-related renal and hepatic damage, measured by classical biochemical markers (AST, ALT, creatinine) and histological evaluation, were reverted by both treatments. Unfortunately, no data regarding inflammatory markers were collected [28].

Another example of an ET-containing plant used in traditional Chinese medicine (TCM) with anti-inflammatory purposes, is *Cleidion brevipetiolatum* Pax & Hoffm (Euphorbiaceae). Zhao et al. isolated six new ETs called brevipetins (B–G) from the ethanolic extract and attributed to brevipetin E (50 mg/kg, p.o.) an anti-arthritis effect in the model of collagen-induced arthritis (CIA). The compound also impaired the NF-κB activity, COX-2, and iNOS expression in LPS-stimulated murine macrophages (RAW 264.7) [29].

Other authors treated CIA mice with a HT-rich fraction from the pericarp of *Terminalia chebula* Retz. fruit, a plant (fam. Combretaceae) belonging to the Ayurvedic tradition. It contained peculiar HTs known as chebulinic and chebulagic acid but also more common ETs such as corilagin, casuarinin, or GTs, such as 1,2,3,4,6 pentagalloyl glucose; 1,2,3,6 tetragalloyl β d-glucose; and 2,4,6 tri-O-galloyl d-glucose. The tannin-rich fraction (100, 200, and 400 mg/kg p.o.) was compared with methotrexate (2 mg/kg) and reduced paw volume, arthritic score, spleen index, and serum cytokine level (TNF-α, IL-1β, and IL-6) in a dose-dependent fashion. All doses impaired TNF-α and IL-1β release and preserved the articular texture at the level of arthritic joints [30]. In line with these results, Lu et al. evaluated the role of pure chebulinic acid administered p.o. (50 mg/kg/d) in collagen-induced arthritis mice, observing a reduction in paw swelling associated with impaired inflammatory infiltration and cartilage erosion at the joint level. The authors also suggested that chebulinic acid may counteract synovial angiogenesis, since VEGFR-related genes were downregulated [31].

The role of corilagin as an individual compound was evaluated in the FCA model (20, 40 mg/kg, p. o.) in comparison to methotrexate. The in vivo results showed that corilagin significantly reduced paw swelling and arthritis score and inhibited joint erosion and the infiltration of inflammatory cells; pro-inflammatory cytokines were also inhibited at the serum level (IL-6, TNF-α, IL-1β, and IL-17). In fibroblast-like synoviocyte (FLS) cells challenged by IL-1β, corilagin (6.25 or 12.5 μM) impaired COX-2, iNOS, and MMPs expression through MAPKs and NF-κB inhibition [32].

Other interesting indications were derived from experiments in which the efficacy of pure EA was investigated, considering the high presence of free EA in pomegranate and its production at the gut level following the metabolization of ETs. Three in vivo studies demonstrated that the oral administration of EA reduced joint swelling and articular disruption in an AIA model [33,34]. The efficacy of EA (50 mg/kg/d) was comparable to the reference compound celecoxib (5 mg/kg). The mechanism of action was attributed to NF-κB impairment and a reduction in MMP-9, VEGF, and TNF-α levels. Moreover, an improvement in oxidative markers was observed, such as MPO activity, NO release, GSH levels, and lipid peroxidation. Similar doses of EA were used by Song et al. (25, 50, 100 mg/kg/d), who confirmed the reduction of paw swelling, synovial hyperplasia, and inflammation in a collagen-induced arthritis (CIA) model [35]. Moreover, EA was treated in TNF-α-induced FLS cells, at the range of 10 to 100 μM, thus inhibiting the levels of cytokines (IL-6, IL-1β) and oxidative markers (MDA). The authors suggested that EA may interfere with the metastasis-associated gene 1 (MTA1)/HDAC-1 pathway, involved in RA development, reflecting the inhibition of Nur77 deacetylation [35].

Despite the different way of administration (i.p.), Allam et al. obtained similar results with EA 175 mg/kg/week in the AIA model. EA significantly inhibited paw edema and reduced serum levels of pro-inflammatory cytokines (TNF-α, IL-1β, IL-17). On the contrary, increased serum levels of IL-10 were measured, thus suggesting a re-balancing of the inflammatory status [36].

Another key metabolite of HTs is GA. Several studies investigated the effect of GA-containing plant extracts in rodent models of arthritis. Polar extracts from *Alternanthera bettzickiana* (Regel) G. Nicholson and *Sarcococca saligna* Müll. Arg., characterized for the presence of GA as one the main compounds (around 0.5 and 1.7 mg/g, respectively), were administered orally at doses of 250, 500, and 1000 mg/kg [37,38]. Both studies reported a reduction in paw swelling and pro-inflammatory cytokines, such as TNF-α, IL-1β, and IL-6. The expression of COX-2 was also reduced, and the molecular docking analysis from Manan et al. suggested that GA might also interfere with enzymatic activity. Moreover, an increase in anti-inflammatory mediators (IL-10, IL-4) and the impairment of the NF-κB pathway were observed.

The anti-inflammatory effect of GA was sustained by another in vivo study in which the pure compound was administered orally (3–100 mg/kg) in pain and arthritis models. In particular, the reduction of TRPA1-mediated edema or allodynic and neuropathic pain was observed at a dose of 10 mg/kg. Accordingly, gallic acid (10 μM) inhibited calcium influx in mouse spinal cord synaptosomes induced by the TRPA1 agonist cinnamaldehyde [39]. A subsequent study confirmed the effect of a GA-rich ethyl acetate fraction (0.01–100 mg/kg) from *Tabernaemontana catharinensis* A. DC. in TRPA1-induced paw inflammation and FCA-induced arthritis. The same fraction potently inhibited TRPA1-induced calcium influx (IC50 = 0.023 μg/mL) but not TRPV1, thus suggesting a specific activity on TRPA1 [40].

From a structure–activity relationship point of view, it is also interesting to note that methyl gallate (0.7 to 70 mg/kg, p.o.) exhibited similar effects with respect to GA in the AIA model. In fact, a dose of 7 mg/kg was enough to reduce leukocyte infiltration, osteoclast activity, and pannus formation at the joint level [41].

An in vitro study suggested that GA might also act at the joint level on synoviocytes: the authors demonstrated that GA (0.1–1 μM) was able to counteract the proliferation of FLS from RA patients through pro-apoptotic mechanisms; moreover, it suppressed the expression of cytokines (IL-1β, IL-6), chemokines (MCP-1, MCP-3), MMP-9, and COX-2 versus the TNF-α challenge [42].

Along with synoviocytes and macrophages, dendritic cells (DCs) play a well-recognized role in the pathogenesis of RA and other inflammatory-based diseases. The role of HTs and their relative metabolites, such as GA, EA, and urolithins (Uro), on this cell population is still unclear. Given the poor bioavailability of unmetabolized HTs, their potential bioactivity might strictly regard resident DCs orchestrating epithelial inflammation, as occurs in psoriasis, AD, and IBDs.

Two in vitro studies investigated the impact of GA or oenothein B treatment on the activation of human DCs: in LPS or cytokine-induced cells, GA (50 μg/mL, corresponding to 300 μM) and oenothein B (10, 25, 50 μM) inhibited the expression of markers with a key role in DCs–T cells interaction, such as CD40, CD80, CD83, and CD86. The effect of GA was also demonstrated in addition to hydrocortisone (0.2 μM), thus suggesting its possible complementary use. GA and oenothein B also inhibited the release of cytokines involved in Th1 polarization, such as IL-12p40 and IL-6, respectively [43,44].

Differently from EA and GA, urolithins were not investigated in models of RA as isolated compounds. However, a recent study attributed anti-inflammatory properties to UroA through in vitro and in vivo experiments, involving surgically induced osteoarthritis; the main mechanism was ascribed to NF-κB pathway impairment [45]. Accordingly, our group reported that urolithins (UroA, UroB, UroA-8Me) at 25 μM can counteract MMP-9 release in human macrophages (THP-1), challenged by TNF-α [46]. Shen et al. managed a work by means of the experimental model of autoimmune encephalomyelitis, suggesting that also urolithin A (UroA) may counteract Th1 inflammation. In fact, UroA (25 mg/kg/d) reduced the activation of DCs and the Th1/Th17 infiltrate at the CNS level. In vitro, UroA (25 μM) inhibited bone-marrow-derived DCs activation and Th17 differentiation in a co-culture model, thus leading to a IL-10 increase and IL-1β, IL-6, and IL-17 reductions, plausibly by acting on the AHR receptor [47].

Despite the concentrations used in several studies, which appear quite high, studies in the literature suggest that HTs may exert an immunosuppressive effect on activated macrophages and dendritic cells after gut metabolization, thus reflecting anti-inflammatory and analgesic effects in animal models of RA (Table 1).

#### 2.1.2. Psoriasis

As previously mentioned, psoriasis and RA are frequently associated in patients with autoimmune disorders, and it is not surprising that psoriatic arthritis often resembles RA symptoms. The cytokine TNF-α is a well-known pathogenetic mediator of many immune-mediated diseases, including psoriasis, targeted by the first biological drugs, such as etanercept and infliximab. However, clinical trials demonstrated the heterogeneous response of psoriasis patients to anti-TNF-α treatments, thus stimulating the search for novel therapeutical targets [48]. In this context, the discoveries regarding the role of IL-23/Th17 axis represent an important advance for pharmacology, as reviewed by Girolomoni et al. [49].

Despite the inhibition of cytokine signaling, including TNF-α, as one of the main bioactivities attributed to HTs [50], few studies have been carried out to elucidate their potential role in psoriasis.

Recently, our research group published an article regarding the potential anti-inflammatory activity of *Rhus coriaria* L. fruit at the skin level, namely a tannin-rich spice from the Mediterranean and Middle-Eastern regions. Water and ethanolic extracts (1–50 μg/mL) from *Rhus coriaria* L. fruit exhibited an inhibitory effect on TNF-α-induced cytokines in human keratinocytes, with IC50s lower than 20 μg/mL. Ethanolic extracts exhibited more promising effects and inhibited MMP-9, ICAM-1, IL-8, and NF-κB-driven transcription. The major compounds identified were GTs (4.54%), followed by a lower content of flavonoids (0.23%) [51]. On the contrary, we excluded the role of the main GT from *Hamamelis virginiana* L., namely hamamelitannin, in the impairment of NF-κB and cytokine release in human keratinocytes challenged by TNF-α [52].

Other authors investigated the biological activity of an ethanolic extract from *Woodfordia fruticosa* (L.) Kurz flower, a source of tannins with traditional uses, from tropical to subtropical areas. The extract was formulated as gold nanoparticles Carbopol^®^ 934 ointment gel (500 μg/kg) or Carbopol^®^ 934 only (2000 mg/kg) and applied topically in a mice model of imiquimod (IMQ)-induced psoriasis [53]. Both formulations reduced the disease score, the serum level of TNF-α and IL-23, and the hyperproliferation of keratinocytes. EA was suggested as the main bioactive compound.

Few works reported the role of ETs in psoriatic inflammation. Punicalagin was administered topically (25 mg/kg) in IMQ-induced mice in comparison to dexamethasone, thus improving psoriasis symptoms. The level of cytokines such as CXCL1, CCL20, and IL-1β was reduced in skin tissue and paralleled NF-κB impairment. Moreover, punicalagin inhibited the release of IL-1β in TNF-α and IL-17A challenged human keratinocytes (HaCaT cells), once again by NF-κB impairment [54]. Similarly, agrimoniin and pedunculagin inhibited enzymatic activity but not the release of human neutrophil elastase ex vivo with low IC50s (<3 μM). The direct interaction of ETs with the enzyme was supported by docking analysis. Moreover, agrimoniin exhibited an anti-proliferative effect in the ATP assay in HaCaT cells (IC50 = 3.4 μM) [55]. To the best of our knowledge, HTs are poorly permeable across skin barrier after topical application and plausibly reach nM concentration at the dermal level [56]; however, μM concentrations might be potentially reached through adequate formulative strategies [57]. The impact of orally administrated ETs and GTs in psoriatic models has rarely been investigated.

According to the literature, the efficacy of HTs and their metabolites on psoriasis has not been fully elucidated. Papers on this subject are limited, thus making it impossible to draw clearcut conclusions (Table 2).

#### 2.1.3. Inflammatory Bowel Diseases (IBDs)

IBDs are represented by ulcerative colitis (UC) and Crohn’s disease (CD). The paradigm of CD and UC as Th1 and Th2 diseases, respectively, was recently criticized by many experts in the field, including Ramos et al. [58]. Since the IL23/Th17 axis became crucial to signaling in the development of novel effective biological drugs, such as ustekinumab, in both IBDs [59,60], we inserted a common paragraph regarding the role of plant extracts in IBDs pathogenesis in this section.

Mango fruit (*Mangifera indica* L.), a well-known source of GTs and free GA, was investigated by several in vivo studies involving UC mice models (DSS-induced colitis). A mango beverage (89.74 mg/kg/day of gallic acid eq.) reduced colon inflammation and cytokine expression (TNF-α, IL-1β, IL-6); moreover, the expression of COX-2 and iNOS and the NF-κB pathway were impaired. The results were sustained by in vitro studies on colon fibroblasts and epithelial cells (CCD-18Co and HT-29 cells, respectively), in which a polar mango extract (10 μg/mL) inhibited iNOS and the mTOR pathway [61]. A mango beverage (475.90 mg/L gallic acid eq.) was compared with a pomegranate beverage (2504.74 mg/L gallic acid eq.) in another study involving a DSS-induced colitis model. Mice received the respective beverage ad libitum (about 90 mL/day) for 3 weeks before colitis induction, showing reduced inflammatory and ulceration scores. Cytokines such as TNF-α, IL-1β, IL-6, and MAPKs were inhibited in the intestinal mucosa; moreover, the mTOR pathway was equally modulated by mango and pomegranate, although different genes were involved in the mechanisms exerted by each beverage. In addition, the expression of IGF-1R and EGFR, known to activate the mTOR pathway, was impaired by mango, pomegranate (10 μg/mL), and the respective major compounds GA and EA (4 μg/mL) in CCD-18Co cells [62].

Of note, Barnes et al. measured relevant levels of GTs and GA metabolites in the urine of healthy volunteers consuming 400 mg/day of mango pulp (*Mangifera indica* L. cv. Keitt) for 10 days. The metabolites pyrogallol-O-sulfate and deoxypyrogallol-O-sulfate were the main metabolites excreted across the considered period, ranging from 28.5 to 55.4 mg and 23.6 to 47.7 mg of total excretion, respectively [1].

Other in vivo studies evaluated the effect of pomegranate peel extract after oral consumption in rodent models of colitis. A decoction of pomegranate peel (300 mg/kg/day), containing 45.3 mg of total ETs, reduced the visceral sensitivity measured by a visceromotor response test. The decoction was compared with the corresponding ETs-enriched fraction (45 mg/kg/day), which was considered responsible for the improvement of visceral pain and the intestinal damage score. Both preparations reduced the infiltration of mast cells and the density of collagen fibers as an index of fibrosis in mucosal stroma [63]. Another work showed that pomegranate extract (250 mg/kg/day), characterized for punicalagin (35%), punicalin (13%), and EA (8.9%) content, caused a slight reduction in the inflammatory infiltrate in DSS-injured intestinal mucosa, while UroA (15 mg/kg/day) showed a statistically relevant inhibitory effect. Both the extracts and UroA prevented the upregulation of COX-2 and iNOS expression, leading to lower NO and PGE2 tissue levels [64].

Pomegranate extract (250–500 mg/kg/die) was compared with pure EA (10–20 mg/kg/die) by Rosillo et al. in a model of TNBS-induced colitis [65,66]. The authors observed similar effects, with reduced neutrophil infiltration and intestinal injury. The anti-inflammatory mechanism consisted of lower iNOS and COX-2 expression and the impairment of MAPKs and the NF-κB pathway.

Two papers investigated the impact of walnut administration in colitis models. The first observed that the addition of 7–14% of walnut to the mouse diet caused partial protection against DSS-induced mucosal damage. The formation of urolithin A and EA was confirmed in fecal samples [67]. In the same model, a phenolic extract from walnut (10–20 mg/kg/day), characterized for the presence of GA and EA, significantly prevented colitis with respect to either acute or chronic DSS-induced damage. In vitro experiments, conducted on intestinal epithelial cells (COLO 205) challenged with TNF-α, attributed an anti-inflammatory effect to walnut phenols (10–20 μg/mL), since NF-κB activity and IL-8 expression were impaired [68].

One author aimed to validate the traditional use of the root bark from *Paeonia* × *suffruticosa* Andrews (called “Moutan Cortex Radicis”) in TCM. The aqueous extract is known to contain a relevant amount of the GT pentagalloyl–glucose (PGG), which was investigated as a main bioactive compound. The oral administration of 5% aqueous extract reduced the inflammation score and the infiltration of macrophages in the intestinal mucosa of DSS-induced mice. PGG (5–10 µM) inhibited NF-κB and IRF in THP-1 cells challenged by TRL2 ligand or poly (I:C) [69].

Similarly, corilagin (7.5–30 mg/kg/day, i.p.) counteracted the shortening of the colon length, caused by DSS induction, in a dose-dependent manner. Moreover, the level of cytokines (TNF-α, IL-1β, IL-6) and MPO activity were reduced in colon tissues [70].

The contribution of pure GA for colitis relief was evaluated by two in vivo studies. In the first, GA (10 mg/kg/day, p.o.) prevented a reduction in body weight and a shortening in colon length and also reduced tissue inflammation. The level of cytokines and MPO was reduced and associated with NF-κB inhibition. In LPS-induced RAW 264.7 cells, high concentrations of GA (100–200 µg/mL, corresponding to 589–1178 µM) decreased p65, iNOS, and COX-2 expression, as well as STAT3 phosphorylation [71]. Accordingly, Zhu et al. showed that GA (20–60 mg/kg, intragastric injection) improved the histological alteration in TNSB-induced colitis, by anti-inflammatory mechanisms which include the NF-κB impairment. In intestinal epithelial cells (HIEC-6) stimulated by IL-1β, GA (20–60 µg/mL) inhibited the cytokines release (IL-6, IL-12, IL-17, IL-23, TGF-β, and TNF-α) and exhibited anti-apoptotic activity [72]. A summary of commented articles is reported in Table 3.

### 2.2. Th2 Inflammation: Asthma, Atopic Dermatitis

**Figure 4:**
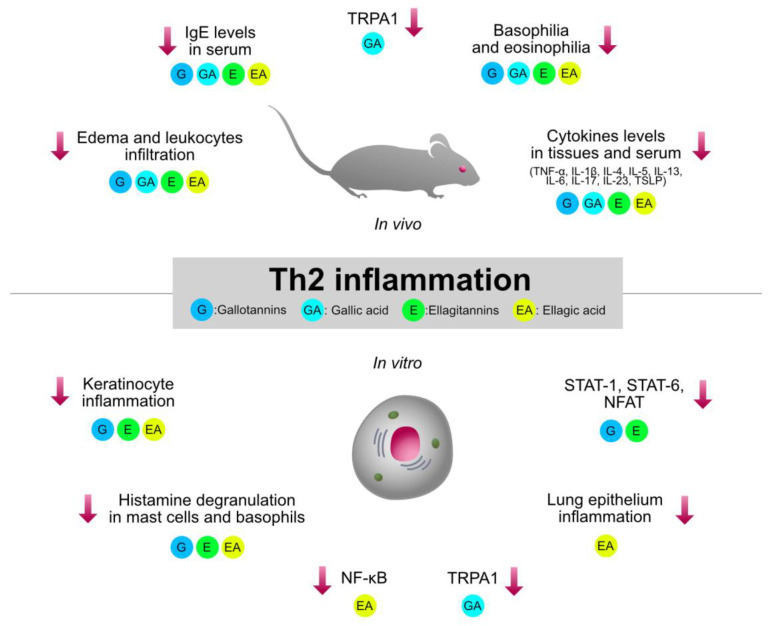
Graphical summary of the anti-inflammatory mechanisms of HTs and their hydrolysis derivatives (GA, EA), investigated in type 2 inflammation models (asthma, AD, allergic rhinitis). The compounds share immunosuppressive properties on innate cytokines (TNF-α, IL-1β, IL-6) and type 2 cytokines (TSLP, IL-4, IL-5, IL-13), which are modulated by the NF-κB and STAT pathways. G, gallotannins; E, ellagitannins; GA, gallic acid; EA, ellagic acid. Red arrows indicate inhibition.

Type 2 inflammation, orchestrated by Th2 cells, plays a crucial role in the pathogenesis of immune-mediated and allergic disorders, such as asthma, AD, and allergic rhinitis. HTs-containing plants and the relative isolated compounds were investigated by several pre-clinical models involving type 2 inflammation. The immunomodulatory mechanisms shared by HTs are summarized in Figure 4.

#### 2.2.1. Atopic Dermatitis (AD)

AD, asthma, and allergic rhinitis are among the most common diseases occurring in children [73]. The complex pathogenesis of asthma and AD were better clarified in recent years, thus suggesting novel therapeutical strategies, such as anti-TSLP (tezepelumab) and anti-IL-4 (dupilumab) antibodies, respectively [74,75]. Asthma and AD are frequently associated with atopy and are characterized by elevated levels of type 2 cytokines (IL-4, IL-5, IL-13), which cause IgE production, mast cell activation, and basophil and eosinophil recruitment, well-known cellular actors in IgE-mediated inflammatory response [76,77]. These recent advances represented winsome tools for the discovery of novel small molecules and the validation of potential anti-allergic natural products.

A plant diffused in the Mediterranean area with traditional usage against mucosal and epithelial inflammation is oak (*Quercus* spp.). Galls occurring in oak are a well-known rich source of GTs, with medicinal and industrial uses in different areas of the world. Tannic acid and tannin-like synthetic polymers (such as Tamol PP) have been used to relieve allergic itch and inflammation [78]. However, the potential role of HTs-rich extracts in allergic diseases is still poorly investigated.

An important effort toward the clarification of AD pathogenesis and the validation of plant traditional medicines was made in Korean research. An ethanolic extract from acorn shells from *Quercus mongolica* Fisch. ex Ledeb. (1% topically), collected in Korea, inhibited the epidermal edema in mice models of oxazolone- and DNCB-induced dermatitis. The level of cytokines involved in AD (TNF-α, IL-1β, IL-33, IL-4) and serum IgE were reduced. The fractionation of the extract correlated with the bioactivity with the EA and GA content. Accordingly, EA and GA were active on IL-4 release and degranulation in RBL-2H3 cells (10–30 μg/mL, corresponding to concentrations < 10 μM) [79]. Similarly, the tannin fraction from common oak bark (*Quercus robur* L.) showed a concentration-dependent inhibition of basophilic degranulation capacity (58–580 μg/mL) in RBL-2H3 cells. Moreover, it inhibited cytokine release (IL-8, IL-6, TNF-α) in human mast cells (HMC-1) [80].

*Quercus* spp. are also known to contain tannic acid, investigated as a pure compound (80 mg/kg/day p.o.) in a NC/Nga mice model of AD induced by *Dermatophagoides farinae* (DfE) cream. Tannic acid strongly lowered the dermatitis score, while moderately inhibiting epidermal thickness and neutrophil infiltration. Several cytokines, such as TNF-α, IFN-γ, and IL-1β, were inhibited at the tissue level, while IL-4, IgE, and IFN-γ were inhibited at the serum level. PPARγ agonism and NF-κB inhibition were involved as potential anti-inflammatory mechanisms [81].

Another important source of GTs is witch hazel (*Hamamelis virginiana* L.), traditionally used to treat skin inflammation and hemorrhoids [82]. Stems and leaves were extracted with ethanol and characterized for the presence of hexagalloylglucose (HGG) as the main compound. The extract (2% in the cell media) inhibited calcium influx induced by particulate matter in human keratinocytes (HaCaT cells). The expression of the PAR-2 receptor and the consequent activation of NF-κB were also inhibited. In addition, the expression of occludin, a protein involved in epithelial integrity, was recovered. HGG (100 μg/mL) resembled the observed inhibition of calcium influx [83].

Recently, our group of researchers attributed an anti-inflammatory activity to a polar extract from witch hazel bark and twigs (0.5–125 μg/mL) in HaCaT cells challenged with TNF-α and IL-4 [52,84]. The extract was characterized by the presence of oligomeric proanthocyanidins (0.29%) and hamamelitannin (0.30%) as the main compounds. The latter (at a concentration lower than 10 μM) showed a specific role in the inhibition of IL-4 targets, such as the release of TSLP and eotaxin-3 (CCL26), and the impairment of cytokeratin-10 (CK-10) and involucrin (INV) expression.

Several articles suggest that GA may represent the anti-allergic moiety of HTs or an anti-allergic molecule itself. GA (0.01–10 μM) inhibited histamine release, and the elevation of [Ca2+]i induced cAMP increase and inhibited NF-κB and MAPK activity in rat mast cells (RPMC) induced by IgE or 48/80. Moreover, it lowered IL-6 and TNF-α after PMA induction in human mast cells (HMC-1). In vivo, GA (1–100 mg/kg) showed 50% inhibition in a IgE-mediated passive cutaneous anaphylaxis model (PCA) and inhibited serum histamine at 10 mg/kg p.o. [85]. Similarly, GA (20–40–80 mg/kg, p.o.) inhibited the TNF-α and IgE serum level in a dose-concentration manner in DNCB-induced mice. The effect was associated with reduced leucocyte infiltration and ear thickness in histopathological evaluation. It also reduced lymph node weight and the expression of TNF-α, IL-4, IFN-γ, and IL-17. In the ear, topical GA reduced the expression of IL-4, IL-5, IL-17, and IL-23, while the expression of IL-10 and TGF-β were increased. In line with this data, the expression of the pro-inflammatory factor ROR-γt was decreased and the suppressor SOCS3 was increased [86].

ETs were even less investigated in AD models. Casuarinin isolated from *Hippophae rhamonoides* L., a plant widely distributed in the Mediterranean region, was investigated in HaCaT cells challenged by TNF-α or IFN-γ. The concentrations of 5–20 μM impaired the proallergic chemokines CCL17 and CCL22, plausibly through STAT-1 and NF-κB inhibition [87]. Of note, the hydrolysis product EA was demonstrated to improve IgE-mediated cutaneous anaphylaxis in SD rats, at doses (10–50 mg/kg, p.o.) comparable with azelastine (10 mg/kg). EA (50–100–200 μM) was also able to impair histamine and cytokine (TNF-α, IL-6) release in ex vivo rat mast cells, by controlling [Ca2+]i and NF-κB activation [88]. EA was also considered the main bioactive compounds in *Rubus coreanus* Miquel root extract (100 mg/kg, topically), which was shown to control the severity of diseases in a DNCB-induced NC/Nga mouse model. The extract had comparable effect to tacrolimus ointment, used as positive control at the same concentration (100 mg/kg, topically) [89]. The summary of commented articles is reported in Table 4.

#### 2.2.2. Asthma and Allergic Rhinitis

The major cause of asthma and allergic rhinitis, in genetically predisposed subjects, is exposure to respiratory allergens [77]. HTs were traditionally used to link and trap allergic aptens to avoid respiratory disorders: environmental sprays based on tannic acid (3%) were investigated by clinical trials for their ability to reduce exposure to typical house allergens in children [90].

However, tannic acid also exhibits pharmacological activity, according to several in vivo studies. The intratracheal application of tannic acid (25 mg/kg) significantly decreased OVA-induced airway hyperresponsiveness in BALB/c mice. The authors observed a reduction of leukocyte infiltration and reduced levels of pro-allergic mediators (Th2 and Th1 cytokines, eotaxin, and total IgE). Moreover, the compound attenuated the expression of mucins (Muc5ac and Muc5b), mucus production in airway goblet cells, mast cell infiltration, and airway remodeling. The mechanism was attributed to the impairment of NF-κB and adhesion molecules at the lung epithelial level [91].

Tannic acid also showed anti-allergic properties after oral administration (40 mg/kg) in an OVA-induced rhinitis model in comparison to dexamethasone (5 mg/kg), based on another study [92]. Tannic acid diminished the number of rushes, histamine and IgE levels, Th2 cytokines (TSLP, IL-4, IL-5, IL-13, IL-33), and innate cytokines (IL-1β, TNF-α). In addition, protein expression levels of caspase-1, MCP-2, and ICAM-1 were reduced. Accordingly, the oral administration of tannic acid (4 mg/mL in water ad libitum) isolated from *Rhus javanica* L. extract showed an anti-allergic effect in a general model of OVA-sensitization (i.p. injection). It lowered IgE production by inhibiting IL-4-induced ε germline transcript (εGT), known to guide the IgE switch [93]. The same authors also investigated the in vitro mechanism of tannic acid (1 μg/mL) in comparison to PGG (1, 10, 25 μM). Both compounds inhibited the IL-4 pathway by acting on IL-4Rα, JAK3, and STAT6 activation in lymphoma cells (DND39) and fibroblasts (NIH3T3). Of note, PGG exhibited the preferential inhibition of IL-4 signaling rather than IFN-γ, thus suggesting selectivity in type 2 inflammation.

The role of PGG was then sustained by an in vivo study, in which oral administration (10 mg/kg) caused a decrease in IgE levels in the serum of OVA-sensitized mice. The production of Th2 (IL-4 and IL-13), Th1 (IFN-γ), and pro-inflammatory cytokines (IL-6, TNF-α), but not anti-inflammatory cytokine (IL-10), from splenocytes was strongly suppressed. In contrast with the observation regarding IL-10 levels, PGG enhanced the proliferation of Treg cells, known for their immunosuppressive properties.

Finally, PGG administration reduced the expression of eotaxin, TIMP-1 (marker of mast cell infiltration), but also increased the expression of IGFBP-3, which inhibits IgE production [94].

Another group evaluated the in vitro bioactivity of eight GTs from *Euphorbia* spp. (*E. jolkini* Bioss and *E. fisheriana* Steud.), showing different sugar moieties and degrees of galloylation (1-O-galloyl-b-D-glucose, 1,2,3-tri-O-galloyl-b-D-glucose, 1,2,3,4,6-penta-O-galloyl-b-D-glucose, 3-O-galloylquinic acid, 2-O-galloyl-D-galactose, 1,3,6-tri-O-galloyl-b-D-allose, 1,2,6-tri-O-galloyl-b-D-allopyanose, and 1,2,3,6- tetra-O-galloyl-b-D-allopyranose). Their effect was evaluated at different concentrations (0.1, 1, 10 μg/mL) in human mast cells (HMC-1) induced by PMA+A23187 (calcium ionophore) in comparison with GA (10 μg/mL), used as positive control. The highly galloylated compounds inhibited the gene expression and secretion of pro-inflammatory cytokines (TNF-α, IL-1β, IL-6) in a concentration-dependent manner, acting on the transcriptional activity of NF-κB [95].

In line with the potential role of GTs, several studies investigated the bioactivity of GA: Fan et al. suggested that the oral administration (20–80 mg/kg) alleviated nasal allergic symptoms in an OVA-induced allergic rhinitis model. The compound decreased the levels of interleukin IL-4, IL-5, IL-13, and IL-17 in nasal lavage fluid and diminished the levels of OVA-specific IgE, IgG1, and IgG2a in serum. In this study, the observed increase in type 1 cytokines (IFN-γ and IL-12) in nasal fluids suggested an inflammatory-state re-balance [96]. Accordingly, GA (10, 50, 100 μg/mL) exhibited suppressor activity on IL-33-induced human basophils (KU812 cells), as is evident from the inhibition of adhesion molecules (ICAM-1) and pro-inflammatory mediators (CCL2, CCL5, CXCL8, IL-6) [97].

The anti-allergic properties of ETs were poorly investigated. An in vivo study demonstrated the anti-inflammatory effect of pomegranate leaf extract (20 mg/kg, intranasal) in a model OVA-induced asthma [98]. The authors suggested the presence of HTs according to HPLC/UV analysis, but their nature was not elucidated. Two additional studies evaluated the role of corilagin: the first reported a decrease in leukocyte and eosinophil counts in the blood of milk-sensitized mice after the oral administration of corilagin (10, 20, 40 mg/kg/day, p.o.); moreover, in another model, corilagin attenuated the anaphylactic reaction, IgE levels, and the degranulation of mast cells. The mechanism of action was also ascribed to the antagonism of Ach- and histamine-induced tracheal contraction ex vivo [99]. In the second, corilagin (10–50 μg/mL) was not able to impair histamine release in rat basophils (RBL-2H3), but the cell line and induction protocol were not comparable with the previous study [100].

Another ET (putranjivain A), occurring in *E. jolkini* Bioss, showed an inhibitory effect in another in vitro study on RBL-2H3 and HMC-1 cells, at a concentration range of 0.1 to 10 μM. The compound inhibited the expression of pro-inflammatory cytokines (TNF-α, IL-6, and IL-4) in IgE-induced or antigen-induced cells, through the impairment of NF-κB and NFAT activity. The effect was also observed at the in vivo level, in which oral administration (10 mg/kg) reduced systemic and cutaneous anaphylaxis, the release of serum histamine, and the expression of the histamine H1 receptor [101].

Finally, three papers investigated the role of EA and EA-rich extracts in allergic models. *Lafoensia pacari* A.St.-Hil. ethanol extract (200 mg/kg, p.o.), a Paraguayan plant belonging to Lythraceae, and isolated EA (10 mg/kg, p.o.) exhibited anti-asthma activity in OVA-induced models. The mechanism was attributed to Th2 cytokine inhibition and reduced granulocyte count. In human bronchial epithelial cells, EA (100 μM) significantly reduced the levels of pro-inflammatory mediators (IL-6, IL-8, and CCL-2) induced by house dust mites [102,103]. Similar effects on asthma were observed by Zhou et al., by treating OVA-induced mice with EA (10 mg/kg, p.o.): the compound was shown to impair lung eosinophilia and Th2 cytokines and to counteract NF-κB activation in the respiratory tissue [104]. A summary of commented articles is reported in Table 5.

## 3. Discussion

Natural products containing HTs have been widely applied for anti-inflammatory purpose by physicians from different countries all over the world, and their bioactivity is sustained by many pre-clinical studies. However, the limited knowledge regarding how they could modulate the immune system limits the rational use.

Several Mediterranean plants are well-known sources of HTs, such as *Rhus* spp. *Punica* spp., and *Quercus* spp., with documented traditional use against different inflammatory disorders. Previous in vitro and in vivo studies sustained the anti-inflammatory effect of HTs and their metabolites on macrophages, fibroblasts, and epithelial cells, which resulted in the inhibition of innate mediators, such as TNF-α, IL-6, IL-1β, PGE2, and MMPs [6,7,8]. The main putative mechanism was ascribed to the impairment of the NF-κB pathway and enzymatic function (i.e., COX-2, MAPKs). Although strongly involved in inflammatory-based diseases, these inflammatory mediators have been mainly investigated in animal models of generic inflammation.

The recent advances regarding the pathogenesis of immune-mediated inflammatory diseases represent an opportunity for the discovery of pharmacological mechanisms beyond innate immunity, such as the modulation of the Th-driven response.

The present review aimed to collect evidence regarding the role of HTs-rich plants in Th inflammatory function, with a particular focus on plants traditionally used. The article collection allowed the summarization of the efficacy of HTs and their gut metabolites (GA, EA, urolithins) in animal models of RA, psoriasis, IBDs, AD, and asthma, which are characterized by the strong involvement of the Th1, Th2, and Th17 responses. The impact on other common autoimmune and inflammatory disorders has been scarcely evaluated by scientific articles: for this reason, no available data were found regarding contact dermatitis, multiple sclerosis, systemic lupus erythematosus, or celiac disease.

Regardless the pathological context, the biological activity of HTs is likely to involve the suppression of both innate and adaptive responses, although the latter has still been poorly investigated. On the other hand, among selected inflammatory-based diseases, psoriasis was poorly considered in pharmacological studies regarding HTs, as already discussed in our previous review in 2020 [7].

The role of gut metabolites in the modulation of specific subsets of Th cells and related cytokines is still unclear, although the increase of Treg response and the reduction of type 1 and type 2 cytokines (such as IFN-γ, IL-17, and IL-4) were both documented by several authors [47,92,94,104]. In this regard, interesting work from Zhang et al. may suggest a potential immunosuppressive effect for urolithin A, regardless the type of T cell [105]. Despite the reduction of cytokines involved in the Th response being observed by many authors, the modulation of the JAK/STAT pathway was rarely investigated. However, few articles suggested that HTs may interfere with STAT1, STAT3, and STAT6 [87,93,103].

The body of literature herein suggests the possibility that HTs may act as mild immunosuppressors, with a generally safe profile after oral or topical administration. Plausibly, products from hydrolysis and metabolism, such as GA, EA, and UroA, participate in the biological effect, although their formation at the gut level has rarely been addressed. This evidence is supported by several in vivo experiments, in which HTs and metabolites were administered as individual compounds through the oral route at comparable doses: EA (50 mg/kg) ameliorated the inflammatory profile in RA models [31,32,33,34,35]; similarly EA, GA, and UroA (10–20 mg/kg) inhibited gut inflammation in IBD models [64,65,66,70,71,72], while EA (10 mg/kg) and GA (20–80 mg/kg) reduced allergic inflammation in AD, asthma, and rhinitis [86,88,96,102]. Remarkably, HTs have frequently been mentioned for direct antibacterial and antiviral activity [106], which may suggest a restrained impact on infection risk.

Several methodological concerns regarding the experimental design of collected articles were underlined during the search process. For example, the route of administration was often neither oral nor topical, which are the most common routes in traditional medicine. Moreover, the doses of extracts and compounds were highly variable among different in vivo studies, thus limiting the potential translation to humans: experiments regarding RA and IBDs were conducted in the broad range of 10 to 1000 mg/kg p.o. On the contrary, doses in the range of 10 to 80 mg/kg were plausibly effective in allergic inflammation models.

The route and dose of administration are extremely relevant issues for any consideration regarding the pharmacokinetic fate, which has been poorly reported in terms of the potential role of gut metabolites or skin-permeable compounds. Another criticism regards several articles in which excessive doses were used, thus leading to implausible biological effects.

## 4. Materials and Methods

The literature was collected from the main database from the biomedical area (MEDLINE) and Google Scholar. The collection was updated in May 2022, and no limit was applied to the year of publication. The search methodology was focused on the detection of articles regarding the role of HTs and inflammatory diseases characterized by type 1 or type 2 inflammation. Consequently, the following two classes of words, linked by using the “and” logic conjunction, were searched in article titles and abstracts:

- Class 1: “tannin”, “hydrolyzable tannin”, “gallotannin”, “ellagitannin”, “gallic acid”, “ellagic acid”, “urolithin”.
- Class 2: “inflammation”, “Th1”, “Th2”, “Th17”, “arthritis”, “dermatitis”, “psoriasis”, “Crohn”, “asthma”, “ulcerative colitis”, “rheumatoid”, “IBD”, “multiple sclerosis”, “lupus”, “celiac diseases”.

The review process included peer-reviewed articles written in English, regardless of the nature of the evidence (in vivo, in vitro, in silico, or clinical trials). All of the articles included a comparison with a reference anti-inflammatory drug. Papers regarding other inflammatory contexts but demonstrating molecular mechanisms of potential interest for inflammatory-based diseases (i.e., cancer, degenerative diseases, etc.) were also included. On the contrary, papers lacking information related to doses, botanical name of plants, or chemical characterization were excluded.

## 5. Conclusions

HTs were shown to counteract either innate and adaptive cytokines in classical type 1 or type 2 inflammatory diseases. However, the evidence was not always homogeneous in terms of doses and routes of administration, which were highly variable among the selected studies, thus limiting general conclusions. RA and IBDs were more investigated than other diseases, such as psoriasis. Moreover, ETs were predominantly evaluated in models of RA, while GTs were predominantly evaluated in models of AD and asthma. These observations might suggest a preferential use of different classes of HTs in different inflammatory contexts, according to traditional use. Of note, most of the pre-clinical studies were driven by ethnopharmacological indications, which represent an empirical filter. For all of these reasons, this review may prompt further investigation regarding the mechanism and the efficacy of HTs in specific inflammatory diseases.

## Figures and Tables

**Table 1:** Collection of recent articles concerning the biological activity of HTs and their metabolites in RA models.

Natural Product	Concentration/Dose	Pre-Clinical Model	Effect ↓↑	Reference
*Alternanthera bettzickiana* (GA)	250, 500, 1000 mg/kg/day/4 week p.o.	Rat, FCA-induced arthritis	↓ edema ↓ TNF-α, IL-6, IL-17 ↓ COX-2 ↓ NF-κB ↑ IL-10, IL-4	[37]
Brevipetin E from *Cleidion brevipetiolatum*	50 mg/kg, acute, p.o.	Mouse, CIA and FCA-induced arthritis	↓ arthritis score	[29]
Brevipetin E from *Cleidion brevipetiolatum*	5–50 μM	Mouse macrophages (RAW 264.7)	↓ COX-2, iNOS ↓ NF-κB	[29]
Chebulinic acid	50 mg/kg/day/2 weeks, p.o.	Mouse, CIA	↓ Arthritis score ↓ VEGFR pathway	[31]
Corilagin	20–40 mg/kg/day/3 weeks, p.o.	Rat, FCA-induced arthritis	↓ Arthritis score ↓ TNF-α, IL-6, IL-1β, IL-17	[32]
Corilagin	6.25, 12.5 μM	Human synoviocytes (FLS cells, ex vivo)	↓ COX-2, iNOS, MMPs ↓ NF-κB	[32]
EA	0.13–0.65% *w*/*w*, of the diet, topical	Rat, CA-induced arthritis	↓ edema and pain	[21]
EA	50 mg/kg/day/20 days, p.o	Rat, FCA-induced arthritis (testis damage)	↓ COX-2, iNOS, MPO ↓ LPO ↑ GSH ↓ NF-κB	[33]
EA	50 mg/kg/day/20 days, p.o	Rat, FCA-induced arthritis	↓ Arthritis score ↓ VEGF, MMP-9 ↓ iNOS, LPO ↑ GSH ↓ NF-κB	[34]
EA	25, 50, 100 mg/kg/3 weeks, p.o	Rat, CIA	↓ Arthritis score	[35]
EA	10–100 μM	Human synoviocytes (FLS cells, ex vivo)	↓ IL-6, IL-1β, MDA ↓ HDAC-1/Nur77 deacetylation	[35]
EA	175 mg/kg/week/4 weeks, i.p.	Mouse, FCA-induced arthritis	↓ edema ↓ TNF-α, IL-1β, IL-17 ↑ IL-10	[36]
GA	3–100 mg/kg, acute, p.o.	Mouse, Cinnamaldehyde (TRPA1)-mediated edema, allodynic and neuropathic pain	↓ edema and pain	[39]
GA	10 μM	Mouse spinal cord (ex vivo), Cinnamaldehyde (TRPA1)-induced	↓ Ca^2+^ influx	[39]
GA	0.1–1 μM	Human synoviocytes (FLS cells, ex vivo)	↓ proliferation ↑ apoptosis ↓ IL-1β, IL-6, MCP-1, MMP-9, COX-2	[42]
GA-rich fraction from *Tabernaemontana catharinensis*	0,01–100 mg/kg, acute, p.o.	Mouse, Cinnamaldehyde (TRPA1)-mediated edema, FCA-induced arthritis	↓ edema and pain ↓ Ca^2+^ influx via TRPA1, but not TRPV1	[40]
HTs fraction from *Terminalia chebula* fruit pericarp	100, 200, 400 mg/kg/day/4 weeks, p.o.	Mouse, CIA	↓ Arthritis score ↓ TNF-α, IL-6, IL-1β	[30]
*Juglans regia* extract (Ets)	900 mg/kg (=10% of the diet)/5 weeks, p.o.	Rat, FCA-induced arthritis	↑ TAC, SOD, CAT ↓ hepatic damage	[28]
*Juglans regia* kernel (Ets)	10% w/w of the diet/5 weeks	Rat, FCA-induced arthritis	↑ TAC, SOD, CAT ↓ hepatic damage	[28]
Oenothein B	1–60 μg/mL	Mouse macrophages (RAW 264.7)	↓ TNF-α, IL-6, IL-1β, iNOS ↓ TRL-2, TLR-4 pathway, but not IFN-γ-induced iNOS ↓ NF-κB (p65, p50)	[26]
*Punica granatum* peel extract (ETs)	1–5% *w*/*w*, topical	Rat, FCA-induced arthritis	↓ edema and pain ↓ leucocyte infiltration ↓ TNF-α, IL-1β	[21]
Punicalagin	10–50 mg/kg/day/4 weeks, i.p.	Rat, CIA	↓ edema ↓ M1 phenotype ↑ M2 phenotype ↑ Arg-1, IL-10 ↓ TNF-α, IL-1β, iNOS ↓ NF-κB	[22]
Punicalagin	10–50 mg/kg/2 weeks, i.p.	Rat, FCA-induced arthritis	↓ edema	[23]
Punicalagin	50 mg/kg/2 weeks, i.p.	Rat, FCA-induced arthritis	↓ edema ↓ TNF-α, IL-6	[24]
Punicalagin	0–50 μM	Human synoviocytes (FLS cells, ex vivo)	↓ IL-1β, IL-6, IL-8, IL-17 ↓ NF-κB	[24]
*Sarcococca saligna* (GA)	250, 500, 1000 mg/kg/day/4 weeks, p.o.	Rat, formaldehyde-induced arthritis	↓ edema ↓ TNF-α, IL-6, IL-17	[38]
Tamarixinin A	12.5, 50 mg/kg/day/2 weeks, sq.i.	Rat, CIA and FCA-induced arthritis	↓ arthritis score	[25]
Tamarixinin A	5–20 μM	Rat peritoneal macrophages (ex vivo)	↓ TNF-α, IL-6, iNOS ↓ NF-κB, p38 MAPK	[25]

FCA, Freund’s complete adjuvant; CIA, collagen-induced arthritis; TAC, total antioxidant capacity; HTs, hydrolyzable tannins.

**Table 2:** Collection of recent articles concerning the biological activity of hydrolyzable tannins and their metabolites in psoriasis models.

Natural Product	Concentration	Pre-Clinical Model	Effect	Reference
Agrimoniin	3.4 μM	Human keratinocytes (HaCaT) cells	↓ proliferation	[55]
Agrimoniin and pedunculagin	<3 μM	Human neutrophil ex vivo	↓ Elastase	[55]
Punicalagin	25 mg/kg/day/7 days, topical	Mice, IMQ-induced psoriasis	↓ Severity score ↓ CXCL1, CCL20, IL-1β	[54]
Punicalagin	2.5–20 μM	Human keratinocytes (HaCaT) cells	↓ IL-1β, caspase-1 ↓ NF-κB	[54]
*Rhus coriaria* L. (GTs)	1–50 μg/mL	Human keratinocytes	↓ MMP-9, ICAM-1, IL-8 ↓ NF-κB	[51]
*Woodfordia fruticosa* L. (EA)	500–2000 μg/kg/day/11 days; topical	Mice, IMQ-induced psoriasis	↓ Severity score ↓ TNF-α, IL-23	[53]

GTs, gallotannins; EA, ellagic acid.

**Table 3:** Collection of recent articles concerning the biological activity of hydrolyzable tannins and their metabolites in IBD models.

Natural Product	Concentration	Pre-Clinical Model	Effect	Reference
Corilagin	7.5–30 mg/kg/day/1 week, i.p.	Mice, DSS-induced colitis	↓ Shortening of colon ↓ TNF-α, IL-1β, IL-6 ↓ MPO	[70]
Ellagic acid	10–20 mg/kg/day/2 weeks, p.o.	Rat, TNBS-induced colitis	↓ Neutrophil infiltration; ↓ Intestinal injury; ↓ iNOS and COX-2 expression	[65,66]
GA	10 mg/kg/day/1 week, p.o.	Mice, DSS-induced colitis	↓ Shortening of colon ↓ Tissue inflammation ↓ Cytokines, MPO	[71]
GA	100–200 µg/mL	Mouse macrophages (RAW 264.7 cells)	↓ p65, iNOS, COX-2 ↓ STAT3	[71]
GA	20–60 mg/kg/day/1 week, i. g.	Mice, TNBS-induced colitis	↑ Ulceration score	[72]
GA	20–60 µg/mL	Intestinal epithelial cells (HIEC-6)	↓ IL-6, IL-12, IL-17, IL-23, TGF-β, TNF-α ↓ Apoptosis	[72]
GA, EA	4 μg/mL	Colon fibroblast (CCD-18Co)	↑ IGF-1R and EGFR	[62]
*Juglans regia* extract (ETs, GTs)	10–20 mg/kg/day/2 weeks, p.o.	Mice, DSS-induced colitis	↓ Acute or chronic damage	[68]
*Juglans regia* extract (ETs, GTs)	10–20 μg/mL	Colon epithelial cells (COLO 205)	↓ NF-κB activity and IL-8 expression	[68]
*Juglans regia* kernel (ETs, GTs)	Walnut kernel 7–14% of the diet/2 weeks, p.o.	Mice, DSS-induced colitis	Partial protection against mucosal damage	[67]
*Mangifera indica* Juice (GTs)	89.74 mg/kg/day/3 weeks of gallic acid eq., p.o.	Mice, DSS-induced colitis	↓ Colon inflammation; ↓ TNF-α, IL-1β, IL-6	[61]
*Mangifera indica* extract (GTs)	10 μg/mL	Colon fibroblast (CCD-18Co), colon epithelial cells (HT-29)	↓ iNOS, mTOR	[61]
*Mangifera indica* juice (GTs)	90 mL/day/3 weeks of 475.90 mg/L gallic acid eq., p.o.	Mice, DSS-induced colitis	↓ Ulceration score; ↓ TNF-α, IL-1β, IL-6 ↓ MAPKs	[62]
*Mangifera indica* or *Punica granatum* extracts	10 μg/mL	Colon fibroblast (CCD-18Co)	↑ IGF-1R and EGFR	[62]
*Paeonia* × *suffruticosa* root bark (GTs)	5 % of aqueous extract/5 days, p.o.	Mice, DSS-induced colitis	↓ Ulceration score; ↓ Macrophage infiltration	[69]
PGG	5–10 µM	Human macrophages (THP-1 cells)	↓ NF-κB, IRF	[69]
*Punica granatum* Juice (ETs)	290 mL/day/3 weeks of 504.74 mg/L gallic acid eq., p.o.	Mice, DSS-induced colitis	↓ Ulceration score; ↓ TNF-α, IL-1β, IL-6, and MAPKs	[62]
*Punica granatum* peel decoction (ETs)	300 mg/kg/day/2 weeks, p.o.	Rat, DNBS-induced colitis	↓ Visceral sensitivity ↓ Infiltration of mast cells ↓ Density of collagen fibers	[63]
*Punica granatum* ETs-enriched fraction	45 mg/kg/day/2 weeks, p.o.	Rat, DNBS-induced colitis	↓ Infiltration of mast cells ↓ Density of collagen fibers	[63]
*Punica granatum* extract (ETs)	250 mg/kg/day/25 days, p.o.	Rat, DNBS-induced colitis	↓ Colon inflammation (slight) ↓ NO, PGE2	[64]
*Punica granatum* extract (ETs)	250–500 mg/kg/day/30 days, p.o.	Rat, TNBS-induced colitis	↓ Neutrophil infiltration; ↓ Ulceration score; ↓ iNOS, COX-2	[65,66]
Urolithin A	15 mg/kg/day/25 days, p.o.	Rat, DNBS-induced colitis	↓ Colon inflammation ↓ NO, PGE2	[64]

GTs, gallotannins; ETs, ellagitannins; GA, gallic acid; EA, ellagic acid; PGG, pentagalloylglucose.

**Table 4:** Collection of recent articles concerning the biological activity of HTs and their metabolites in AD models.

Natural Product	Concentration/Dose	Pre-Clinical Model	Effect	Reference
Casuarinin (from *Hippophae rhamonoides*)	5–20 μM	Human keratinocyte (HaCaT cells)	↓ CCL17, CCL22 ↓ NF-κB, STAT-1	[87]
EA	1–50 mg/kg, acute, p.o.	Rat, IgE-induced PCA	↓ mortality	[88]
EA	50–100–200 μM	Rat mast cells (RPMC cells)	↓ TNF-α, IL-6, histamine ↓ NF-κB, Ca^2+^ influx	[88]
EA, GA	10–30 μg/mL (<10 μM)	Rat basophil (RBL-2H3 cells)	↓ IL-4, degranulation	[79]
GA	0.01–10 μM	Rat mast cells (RPMC cells)	↓ Ca^2+^ influx, histamine ↑ cAMP ↓ NF-κB, MAPK	[85]
GA	0.01–10 μM	Human mast cells (HMC-1 cells)	↓ TNF-α, IL-6	[85]
GA	1–100 mg/kg, acute, i.p.	Mouse, 48/80- or IgE-induced PCA	↓ mortality, serum histamine	[85]
GA	20–40–80 mg/kg, 5 weeks, p.o.	Mouse, DNCB-induced dermatitis	↓ dermatitis score ↓ Serum TNF-α, IgE ↓ TNF-α, IFN-γ, IL-4, IL-17, IL-23 ↑ TGF-β, IL-10 ↑ SOCS3, ↓ ROR-γt	[86]
*Hamamelis virginiana* bark and twigs glyceric extract	0.5–125 μg/mL	Human keratinocyte (HaCaT cells)	↓ TSLP, IL-6, CCL26 ↑ CK-10, INV ↓ proliferation ↓ NF-κB	[84]
*Hamamelis virginiana* stems and leaves ethanol extract	2%	Human keratinocyte (HaCaT cells)	↓ Ca^2+^ influx ↓ PPAR-2, ↓ NF-κB ↑ occludin	[83]
HGG	100 μg/mL	Human keratinocyte (HaCaT cells)	↓ Ca^2+^ influx	[83]
HT	1–10 μM	Human keratinocyte (HaCaT cells)	↓ TSLP, CCL-26 ↑ CK-10, INV ↓ proliferation	[84]
*Quercus mongolica* acorn shell, ethanol extract	1%/4 weeks, topical	Mouse, oxazolone- and DNCB-induced dermatitis	↓ TNF-α, IL-1β, IL-33, IL-4, ↓ Serum IgE	[79]
*Quercus robur* bark, tannin fraction	58–580 μg/mL	Rat basophil (RBL-2H3 cells) Human mast cells (HMC-1 cells)	↓ IL-8, IL-6, TNF-α, degranulation	[80]
*Rubus coreanus* root extract (EA)	100 mg/kg/4 weeks, topical	Mouse, DNCB-induced dermatitis	↓ dermatitis score ↓ IL-4, IL-5, IL-12, IFN-γ, TNF-α, TARC, ↓ IgE	[89]
*Rubus coreanus* root extract (EA)	10 μg/mL	Human mast cells (HMC-1 cells)	↓ β-Hexosaminidase, histamine	[89]
TA	80 mg/kg/day/2 weeks, p.o.	Mouse, DfE-cream-induced AD	↓ Dermatitis score ↓ TNF-α, IFN-γ, IL-1β ↓ Serum IL-4, IFN-γ ↑ PPARγ, ↓ NF-κB	[81]

EA, ellagic acid; GA, gallic acid; TA, tannic acid; HGG, hexagalloylglucose; Dfe, Dermatophagoides farinae; HT, hamamelitannin.

**Table 5:** Collection of recent articles concerning the biological activity of HTs and their metabolites in asthma and allergic rhinitis models.

Natural Product	Concentration/Dose	Pre-Clinical Model	Effect	Reference
8 GTs isolated from *Euphorbia* spp.	0.1, 1, 10 μg/mL	Human mast cells (HMC-1 cells)	↓ TNF-α, IL-1β, IL-6 (highly galloylated compounds > others) ↓ NF-κB	[95]
Corilagin	10, 20, 40 mg/kg/day/1 week, p.o	Rodent and guinea pig models, 48/80-induced PCA and milk sensitization	↓ leucocyte and eosinophil counts ↓ mast cell degranulation ↓ PCA reaction, IgE ↓ Ach- and histamine-induced tracheal contraction	[99]
Corilagin	1–50 μg/mL	Rat basophil (RBL-2H3 cells)	No effect on histamine release	[100]
EA	10 mg/kg/22 days, p.o.	Mouse, OVA-induced asthma	↓ Th2 cytokines ↓ granulocytes count	[102]
EA	100 μM	Human bronchial epithelial cells (HBEpC cells)	↓ IL-8, IL-6, CCL-2	[103]
EA	10 mg/kg, acute, p.o.	Mouse, OVA-induced asthma	↓ lung eosinophilia ↓ Th2 cytokines ↓ NF-κB	[104]
GA	20–80 mg/kg/day/12 days, p.o.	Mouse, OVA-induced rhinitis	↓ IL-4, IL-5, IL-13, IL-17 ↓ Serum IgE, IgG1, IgG2a ↑ IL-12, IFN-γ	[96]
GA	10, 50, 100 μg/mL	Human basophil (KU812 cells)	↓ ICAM-1, CCL2, CCL5, CXCL8, IL-6	[97]
*Lafoensia pacari* ethanol extract (ETs)	200 mg/kg/day/22 days, p.o.	Mouse, OVA-induced asthma	↓ Th2 cytokines ↓ granulocytes count	[102]
PGG	1–25 μM	DND39 cells NIH3T3 cells	↓ IL4Rα, JAK3, STAT6	[93]
PGG	10 mg/kg/day/28 days, p.o.	Mouse, OVA- sensitization	↓ Serum IgE ↓ IL-4, IL-13, IFN-γ, IL-6, TNF-α (splenocytes) ↓ TIMP-1, eotaxin ↑ IGFBP-3 ↑ Tregs	[94]
Putranjivain A	1–10 μM	Rat basophil (RBL-2H3 cells) Human mast cells (HMC-1 cells)	↓ TNF-α, IL-4, IL-6 ↓ NF-κB, NFAT	[101]
Putranjivain A	10 mg/kg, acute, p.o.	Mouse, IgE-induced PCA and 48/80-induced systemic anaphylaxis	↓ H1R and histamine ↓ anaphylaxis	[101]
TA	25 mg/kg, acute, i.t.	Mouse, OVA-induced asthma	↓ Th2, Th1 cytokines ↓ adhesion molecules ↓ Serum IgE ↓ Mucus production, Muc5ac, Muc5b expression ↓ NF-κB	[91]
TA	40 mg/kg/day/10 days, p.o.	Mouse, OVA-induced rhinitis	↓ rushes ↓ serum IgE ↓ histamine, TSLP, IL-4, IL-5, IL-13, IL-33 ↓ IL-1β, TNF-α, MCP-2 ↓ ICAM-1	[92]
TA	4 mg/mL/17 days (drinking water), ad libitum	Mouse, OVA sensitization (i.p.)	↓ Serum IgE ↓ IL-4 induced εGT	[93]
TA	1 μg/mL	Human Burkitt lymphoma (DND39 cells) Mouse fibroblast (NIH3T3 cells)	↓ IL4Rα, JAK3, STAT6	[93]

EA, ellagic acid; GA, gallic acid; TA, tannic acid; PGG, pentagalloylglucose; Dfe, Dermatophagoides farinae; HT, hamamelitannin.
